# “We’re Just Tired”: Influences on Sexual Activity Among Male-Partnered Women in Midlife; A Mixed Method Study

**DOI:** 10.1080/00224499.2023.2165613

**Published:** 2023-02-09

**Authors:** Kaye Wellings, Lorna Gibson, Ruth Lewis, Jessica Datta, Wendy Macdowall, Kirstin Mitchell

**Affiliations:** a Public Health & Policy, London School of Hygiene and Tropical Medicine, London, UK; b Department of Social and Environmental Health Research, London School of Hygiene and Tropical Medicine, London, UK; c MRC/CSO Social & Public Health Sciences Unit, University of Glasgow, Glasgow, UK; d Social and Environmental Research, London School of Hygiene and Tropical Medicine, London, UK; e Department of Public Health, Environments and Society, Faculty of Public Health and Policy, London School of Hygiene and Tropical Medicine, London, UK; f MRC/CSO Social and Public Health Sciences Unit, University of Glasgow, Glasgow, UK

## Abstract

Studies into decline in sexual activity among women in midlife produce equivocal findings, some implicating hormonal and physiological changes, others psycho-social and environmental factors. Women’s perspectives rarely inform interpretation of the data. Associations between sexual satisfaction, activity and function, and health and lifestyle factors were explored using data from 2133 female participants in the third British National Survey of Sexual Attitudes and Lifestyles (2010–2012). Semi-structured interviews (2012–2015) with 23 women aged 45–59 reporting sexual dissatisfaction in Natsal-3 explored their perceptions of the influences on their sexual activity. Analysis of the survey data showed sexual dissatisfaction to be less common than low frequency and function. Neither menopausal stage nor age was independently associated with any of the dimensions of sexual experience. Only relationship unhappiness was independently associated with all three and communicational difficulty with two (dissatisfaction and lower function). In-depth interviews identified influences on sexual activity not captured in the survey. Tiredness attributed to contemporary challenges of midlife was a dominant theme. Relationship quality mediated its adverse impact. Sexual experience in midlife must be interpreted in light of both life-stage and era, notably, the increasing demands on women in contemporary society and their impact on vitality. Efforts to address sexual wellbeing should take account of the wider social context.

Sexual activity is recognized as important to quality of life throughout the life course (WHOQOL Group, 1994) and by many as a critical issue in mid-life. Although the majority of men and women aged over 50 are sexually active, both the frequency of sexual activity and sexual function tend to decline with age (Field et al., 2013; Hayes & Dennerstein, 2005; Lindau et al., 2010; Mitchell et al., 2013). The decline occurs earlier and is steeper for women than men and the gender gap widens with age, most markedly in mid-life (Beutel et al., 2008; Burghardt et al., 2020; Field et al., 2013; Lindau et al., 2010).

Understanding the influences on sexual wellbeing in mid-life is important in enabling women to make sense of their experience and to help practitioners provide appropriate advice to those seeking help with sexual problems (Davison et al., 2009). Singling out the main reasons for the decline in sexual activity and function is, however, made difficult by the confluence of potential influences on sexual experience in midlife, particularly among women (Grundy & Henretta, 2006; Lachman, 2004). Biomedical factors include hormonal changes associated with the menopause resulting in vasomotor and urogenital symptoms, mood change, and sleep disturbance in some women (Bancroft & Cawood, 1996; Cintron et al., 2017; C. M. Mitchell et al., 2021). The onset of physiological aging brings changes, which may include weight gain and alterations to self–perceived sexual attractiveness (Thomas et al., 2019). Changes in the physical and mental health status of women or their partners in midlife have the potential to impact on quality of life and sexual function (Bromberger & Kravitz, 2011; Campbell et al., 2020).

Social changes relating to life stage and lifestyle in mid-life may also play a part. For some, work commitments increase at this time of life; for others, job insecurities arise. Children living at home are often in the challenging stages of adolescence, and social and economic changes have increased the possibility that adult children remain for longer in the family home, adding the “full nest syndrome” to the more familiar “empty nest syndrome” in mid-life (Dennerstein et al., 2002; Abreu., 2022; Mitchell & Lovegreen, 2009). Aging parents increasingly need support and those who have died may leave a gap in help previously provided (Lin & Burgard, 2018; Winterich & Umberson, 1999). And more middle-aged women than men are without a sexual partner, since patterns of age mixing in sexual relationships and differential longevity result in gender imbalance in the availability of sexual partners at this time in life (Lindau et al., 2010).

The literature is equivocal on which of these factors contribute most to the decline in sexual activity and satisfaction. Differences in emphases partly reflect the prior hypotheses of researchers, and hence the methodological approach adopted, for example, the choice of setting, the size and composition of the sample, and the variables included in the study. Studies focusing predominantly on biomedical factors have typically taken the menopause as their starting point, often using small clinical samples of patients seeking treatment for menopausal or other gynecological conditions. Such studies have typically been conducted in settings where reasons for attending may include sexual problems and where opportunities for measuring the broader range of factors operating at midlife may be limited. Not surprisingly, their hypotheses have tended to be confirmed and many, though not all (Dąbrowska-Galas et al., 2019), have shown strong associations between the quality and frequency of sexual experience and the menopausal transition (Chedraui et al., 2012; Kong et al., 2019; Ornat et al., 2013; Simon et al., 2018; Valadares et al., 2008; Verit & Billurcu, 2009).

Larger, population-based studies carried out at community level have taken account of a broader range of candidate explanatory variables, including psychosocial and environmental factors. Yet even these studies have produced inconsistent findings. Some have found no independent effect of the menopause, showing instead characteristics such as health status, relationship factors, and social representations of sexuality in mid-life to be stronger influences on sexual activity (Davison et al., 2009; Ringa et al., 2013), while others have suggested an age-related decline in sexual function and frequency, and a further incremental decline associated with the menopausal transition (Dennerstein et al., 2003; Nazarpour et al., 2018).

Studies offering greatest promise in disentangling the variables associated with changes in women’s sexual experience in midlife are those using a longitudinal, cohort design. Such studies have shown factors other than menopausal status and age to be more significant in influencing sexual function and activity in mid-life. Avis et al. (2018), for example, reporting findings from the large, longitudinal SWAN study in the US, found menopausal status to be unrelated in adjusted analyses to arousal, frequency of sexual activity, physical pleasure, or satisfaction with partner. The most significant correlates of these outcomes were the importance of sex to the participant, relationship factors, and mental and physical health. Dennerstein et al. (2008), reporting on a similar longitudinal study in Australia, found only a marginal effect of the menopausal transition on sexual function; relationship quality, paid work, and interpersonal stress were more significant predictors. Prospective longitudinal studies are better able to overcome the difficulties of inferring causality inherent in cross-sectional studies. Yet even so, with some notable exceptions (for example, Burleson et al., 2007), they have been limited in the extent to which they could establish causal direction in the associations revealed, for example, between depression and quality of sexual experience (Jackson et al., 2019; Gracia et al., 2018).

Ultimately, associations can be shown only for variables measured, and those included in studies are generally the ones considered by the investigators to be of likely predictive value. Ideally, the views of women themselves on what factors impact their sexual experience in midlife should guide hypotheses (Harder et al., 2019; Hinchliff et al., 2010; Winterich & Umberson, 1999). Yet qualitative research has rarely informed the design of quantitative studies and even more rarely been reported alongside survey findings. Further, researchers have paid more attention to *which* factors influence sexual experience than *how* they do so (Jackson et al., 2019). Few studies have been designed to explore the mechanisms and pathways in the associations between features of midlife and dimensions of sexual experience.

In this paper, we report findings from the third British National Survey of Sexual Attitudes and Lifestyles (Natsal-3) (https://www.natsal.ac.uk/natsal-survey/natsal-3), and from indepth interviews with women aged 45 to 59 who reported in the survey being dissatisfied with their sex lives. Our aim in analyzing the survey data was to examine the relative strength of influence on aspects of sexual experience in midlife, of biological and hormonal factors, on the one hand, and social and lifestyle factors on the other. Our hypothesis was that the influence of social contextual factors on sexual experience in midlife may be as great as, if not greater than, that of hormonal factors. The research question used to interrogate the survey data was, therefore, which variables – from among a range including hormonal status, measures of physical and mental health, relational features, and lifestyle characteristics – appear to be most strongly associated with different dimensions of sexual experience, i.e. sexual function, sexual frequency, and satisfaction with sex.

Our aims in complementing the survey data with qualitative research among women reporting dissatisfaction with their sex lives, were to a) amplify and explore the meaning of the associations found in the survey data; b) explore women’s own understanding of the influences on their sexual experience in midlife; c) gain insights into the direction of causality in the associations found in the statistical analysis, and d) identify explanatory variables, which are missing from the Natsal data set and yet appear to be important for the women themselves in understanding the impact of aspects of midlife for different dimensions of sexual experience.

## Method

### Survey Component

#### Study Design and Participants

Data were analyzed from the third National Study of Sexual Attitudes and Lifestyles (Natsal-3), a survey of 15,163 men and women aged 16–74 years living in Britain carried out between 2010 and 2012. A multistage, clustered and stratified probability sample design was used. Participants were interviewed in-home using a combination of computer-assisted personal interviews (CAPI), and computer-assisted self-interviews (CASI) for more sensitive questions (including those on sexual satisfaction, frequency, and function). The estimated response rate was 57.7%, and the cooperation rate (the number of interviews completed from eligible addresses for which contact was made) was 65.8%. Details of the methods are described elsewhere (Erens et al., 2014). Eight thousand eight hundred and sixty-nine women took part in Natsal-3, of whom 2228 were aged 40 to 59 at interview, selected for statistical analysis in this study to compare women of pre-, peri-, and post-menopausal status.

#### Outcomes

The outcomes included in the current study were sexual function, sexual satisfaction, and sexual activity, the aim being to measure multiple facets of sexual experience. Sexual function was assessed using the Natsal-SF, a psychometrically validated 17 item (16 items per gender) measure comprising three components. The first includes problems with sexual response, the second captures sexual function in the relationship context and the third, self-appraisal of sex life. Respondents who had at least one sexual partner in the year prior to interview were given a score on the Natsal-SF. Those in the lowest quintile of the sex-specific distribution were considered to have “lower” sexual function (see Mitchell et al., 2012; Jones et al., 2015 for details of the measure and its scoring). For sexual satisfaction, respondents were asked to think about their sex life in the past year and to express their agreement/disagreement with the statement “*I feel satisfied with my sex life*.” on a five-point Likert scale (1. Agree strongly to 5. Disagree strongly). Women were categorized as currently sexually inactive if they answered zero to the question: *“On how many occasions in the last 4 weeks have you had vaginal intercourse/oral sex/anal sex with a man/woman?.”*

In the statistical analysis, all three outcomes were dichotomized. We imposed a categorical measurement on scores on the continuum of sexual function, treating the lowest quintile of the sex-specific population distribution of scores as lower sexual function (i.e. low relative to the rest of the sample). For satisfaction, responses expressing disagreement or strong disagreement were categorized as dissatisfied versus the three alternative responses. Current sexual activity was also treated in binary form, i.e. zero occasions of sex in the past month versus one or more.

Independent variables were grouped into domains of menopausal status, physiological and health-related measures, relational factors and social and lifestyle characteristics. Menopausal status was categorized as pre-menopausal (menses within the past month); peri-menopausal (last menses between 6 and 12 months ago), and post-menopausal (amenorrheic for over a year) with a further distinction by use of HRT (Hale & Burger, 2009). Physiological and health-related measures included self-reported health status (fair, bad, or very bad vs good or very good); depression (measured via the two-item PHQ2 (Arroll et al., 2003) and Body Mass Index (BMI). Relational factors were measured by the status of most recent sexual partner (cohabiting; steady; previously steady; just met); satisfaction with that relationship (degree of happiness); and ease of communication within the relationship about sex. Indicators of social position included educational attainment (graduate vs non-graduate status) and area-level deprivation, using the Index of Multiple Deprivation (Payne & Abel, 2012). Lifestyle factors included women’s employment status and that of her most recent partner; number of children and age of the youngest child. We added two attitudinal variables based on emerging findings from analysis of the first 10 in-depth interviews, derived from Natsal-3 survey questions: *“It is natural for people to want less sex as they get older”* and *“Men have a naturally higher sex drive than women*,” combining responses “agree” and “agree strongly.”

#### Statistical Analysis

Prevalence estimates for each of sexual dissatisfaction, (current) sexual inactivity and lower sexual function were calculated and their associations with the range of factors, including age, menopausal stage, health and relationship status, demographic and lifestyle factors, were explored in bi-variate analysis and subsequently in regression analysis. Survey variables were initially selected for analysis according to hypotheses based on previous analysis of the data (Field et al., 2013; Mitchell et al., 2013) and a review of the literature. All variables showing statistically significant associations and, *a priori*, age and menopausal status, were included in the final regression model. All analyses used the survey commands in STATA (version 14.2) incorporating the weighting, clustering, and stratification of the Natsal-3 dataset. Logistic regression was used to estimate age-adjusted odds ratios (aAOR) and fully adjusted odds ratios (AORs) to explore factors associated with the three outcomes.

### Qualitative Component

#### Study Design and Participants

Eighty-four percent of Natsal-3 participants agreed to be recontacted for an in-depth interview. Twenty-three women were selected who had disagreed with the statement ‘*I feel satisfied with my sex life”*, representing different areas of the country, different ages within the range 46–59 years, and menopausal and post-menopausal stages. Selection was also guided by the recency with which the survey had been completed. Women were re-contacted by one of the researchers, informed of the criteria on which they had been selected, and asked whether they agreed to take part. Recruitment stopped at the point at which themes represented in the accounts had reached saturation. The women were assured that the inter-viewer had seen none of their survey responses apart from the selection criteria. Interviews were carried out face-to-face in women’s homes, recorded with their agreement and transcribed verbatim. The topic guide probed perceptions of middle age; factors influencing the frequency and quality of sexual activity; attitudes toward aging and sexual activity; and the importance and meaning of sex at this time in life. Following preliminary statistical analysis of the survey data, the topic guide was revised to allow associations emerging to be fully explored.

#### Qualitative Analysis

Data were analyzed using a modified Framework Method (Ritchie et al., 2013). Through familiarization with the data, we identified and categorized key themes emerging from the data. We began by identifying themes established *a priori* from the study’s hypotheses, adding inductive codes grounded in spontaneously recurring themes in participant responses. Two researchers (KW and KM) independently coded the transcripts across a subsample of 10 transcripts, following which discrepancies were discussed and resolved, and an agreed coding frame was developed which was used to chart the material from the remaining 13 transcripts on Excel sheets. The jointly agreed codes were systematically applied to the whole data set and new codes were added iteratively as they emerged. A matrix was generated comprising rows (cases), and columns (codes), the resulting cells providing a structure into which the data were systematically entered to analyze by case and by code. Field notes were used to contextualize accounts.

## Results

### Findings from the Survey Data

Two thousand one hundred and thirty-three survey participants were eligible for analysis in the study, being aged 40–59, having ever had sex with a man and having answered all relevant questions. Current sexual inactivity was reported by 35.6% of women; 23.3% were categorized as having lower sexual function; 16.2% were dissatisfied with their sex lives.

#### Age and Menopausal Status

An age-related increase was seen in the prevalence of current sexual inactivity (from 27.1% among 40–44 year olds to 51.6% among 60–65 year olds), but differences were only significant for women aged 60–65 compared with those aged 40–59 (Table 1). No significant differences across age groups were seen for either lower sexual function or dissatisfaction. Menopausal status was not significantly associated with any of the three sexual experience outcomes.

#### Physiological and Health Status

Associations between all the health-related variables and the three outcomes were statistically significant in bivariate analysis (Table 1) and strongest for mental health. After adjusting for age, odds of sexual dissatisfaction, and current sexual inactivity were both twice as high among women with recent experience of depression and for lower sexual function the increase was fourfold (Table 2). Age-adjusted odds of sexual inactivity and lower sexual function were significantly higher among women assessing their health as poor, and age-adjusted odds of all three outcomes were higher among women who were overweight.

#### Relational Factors

Relationship status, happiness in the relationship, and ease of talking about sex, were strongly associated with all three outcomes. Age-adjusted odds for sexual dissatisfaction, current inactivity, and lower function were all higher among women with no live-in partner compared with those cohabiting (Table 2). Those who were unhappy in their relationship had double the odds of being sexually inactive; more than double the odds of being categorized as having lower sexual function; and nearly three times the odds of being dissatisfied with their sex lives. Associations with ease of sexual communication were even more marked. Age-adjusted odds for both sexual dissatisfaction (aOR 5.5^1^) and lower sexual function (aOR 11.9) were markedly higher among women who found talking to their partner about sex difficult, and appreciably higher for sexual inactivity (OR a3.2).

#### Social and Lifestyle Factors

Differences by socio-economic status across the outcomes were nuanced. Graduates had higher odds of being dissatisfied with their sex lives than non-graduates. Women living in poorer areas had higher odds of reporting sexual inactivity than those in more affluent areas (Table 2). Age-adjusted odds for sexual inactivity were nearly twice as high among women who themselves were unemployed and three times as high for those whose partner was unemployed (Table 2). Similar effects were seen for hours worked. There was no significant association between the length of women’s working week and any of the outcomes but age-adjusted odds for sexual dissatisfaction, sexual inactivity and lower sexual function were higher, though not significantly so, among those whose partner worked less than a 35-hour week. Childless women had higher odds of being sexually dissatisfied (Table 2) compared with those with children of any age. Weaker associations were found with attitudinal variables, but odds for having lower sexual function were higher, and for sexual inactivity somewhat higher, among women who believed sexual desire decreased with age.

#### Multivariate Regression Analysis

After full adjustment, (Table 3) only relationship unhappiness remained independently associated with all three outcomes – sexual dissatisfaction (AOR: 2.86 CIs: 1.88–4.35; *p* = <0.0001^1^), sexual inactivity (AOR: 1.59 [1.15–2.19]; *p* = .0050) and lower sexual function (AOR: 2.22 [1.62–3.04]; *p* = .0072). Difficulty in talking about sex remained strongly associated with sexual dissatisfaction (AOR: 4.39 [2.03–9.50]; *p* = .0004) and even more strongly with lower sexual function (AOR: 12.30 [6.18–24.49]; *p* = .0001). Odds of sexual dissatisfaction were higher among graduates than non-graduates (AOR: 1.74 [1.17–2.61]: *p* = 0069). They were higher among women with a child aged under 12 compared with those who were childless (AOR: 3.61 [1.40–9.29] for a child under 5; AOR: 2.36 [1.12–4.96] for a child aged 6–11; *p* = .0449). Odds of having lower sexual function were higher among women self-reporting poorer health status (AOR: 1.53 [1.01–2.32]; *p* = .0431) and among those diagnosed with current depression (AOR: 3.55 [2.21–5.70]; *p* = .0001).

### Findings from the Qualitative Research

Findings from the interviews with women reporting sexual dissatisfaction in the survey are organized firstly by the domains corresponding to significant associations in the survey data and secondly, by themes spontaneously occurring in women’s accounts.

#### Age and Menopausal Status

Aspects of midlife described by women as impacting their sex lives reflected the survey findings with respect to age and menopausal status. For seven women age, in itself, was not seen as a major determinant of sexual activity and satisfaction except in so far as health conditions had deteriorated with age and this had affected their own sexual response or that of their partner. Similarly, few women linked menopausal status to a decline in the quality or frequency of sexual activity. Most were not unduly bothered by menopausal symptoms and, aside from night-sweats and hot flushes, had experienced no adverse physical effects, nor any change in their sex drive. “*Hideous sweating but that’s about it.… interest in sex, that’s the same.”* [1] Some saw benefits of the menopause in terms of freedom from menstrual periods and the risk of pregnancy. “ *… the chances of getting pregnant are virtually nil aren’t they? So from that point of view I look at it in a positive light.”* [5] Only two women directly attributed their lack of interest in sex to the menopause and, for one, the impact was compounded by other factors, onset having coincided with depression following a bereavement, a recent injury and starting to work night shifts [11]. In this instance, depression had preceded the decline in the quality of her sex life. For others, the reverse was true; depression appeared to be consequent on the decline.

#### The Perceived Impact of Lifestyle and Life Stage

Changes to sexual experience in midlife were interpreted less in terms of physiological decline and ebbing hormones, and more in terms of life stage. For those with a long-term partner, advancing years had implications for relationship duration, which was seen as a salient factor determining sexual activity. At this time in their lives, few women were in the first flush of their relationship and familiarity was seen by some as having dulled desire. The accounts of three women – separated from long-term partners and in new relationships, for whom sex had improved with age – lent support to this view.

Midlife was described as a “half-way mark,” and views on what lay ahead were mixed. Some saw the outlook as positive *(“I feel OK about this stage in life. I feel more balanced and in control”*) [15); others saw it as less auspicious. *(“…you’ve had your youth and things to look forward to. From 50, it’s a bit of a downhill spiral*….“ [3]” *I am going through the menopause but it hasn’t really bothered me, what slightly bothered me was turning 50.…Now I’m thinking, am I going start to go downhill?*”. [13) Unlike other researchers (Banister, 2000; Ringa et al., 2013), we found few references to negative social representations of age and sexuality; only two women spoke of their fears of losing physical attractiveness, of “*becoming invisible”* [1].

#### The Relationship Context

The strong association in the survey data between relational factors and the quality of women’s sex lives was echoed in their accounts. Whilst it was not apparent from the survey data whether the quality of the relationship influenced that of the sexual experience, or vice versa, the in-depth interviews suggested that the two were mutually reinforcing. Desire for sex depended on how well women were getting on with their partner but having sex improved this.

I. What about the other way round, what impact does having sex have on your mood?

P. You definitely feel brighter; ‘Oh yeah, we shagged this morning’ (laughs). I suppose yeah, absolutely, you feel good about yourself, don’t you, you feel attractive, it’s like a feeling of belonging to something special. Relationships are special. [2]

Two women reported sexual inactivity having led to a deterioration in the relationship to the point of separation.

Where there were sexual difficulties, talking about them was not seen as easy, especially – according to women’s accounts – for men. Some described their partner’s extreme reluctance to discuss sexual problems. In other scenarios described, couples had neither time nor opportunity to discuss sex, topics of more pressing importance taking priority.

Sex was seen as important to relationships for the opportunity it presented for physical closeness. Nevertheless, where the relationship had retained intimacy, however this was expressed, sexual inactivity mattered less. This may explain the weaker association in the survey data between happiness in the relationship and sexual frequency, compared with that with sexual dissatisfaction.

Women with a partner saw their relationship as protective in terms of the negative impact of midlife events, as a buffer against the demands and stresses of modern life, helping to explain the independent associations in the survey data between relationship happiness and all three dimensions of sexual experience.

#### The Impact of Midlife Pressures

What the survey had failed to capture, and women’s accounts illustrated vividly, was the sheer weight of pressures on women in midlife. Near unanimously, those we spoke to described the complexity of their daily lives at this time, their hectic schedules, and the challenges of combining family, work, and social lives. They talked about financial and relationship difficulties, worries about family members, the simultaneous demands of children and aging parents, both needing practical help and emotional support and neither contributing greatly to a reduction in workload or stress. The “double caring duties” for children and parents were seen as an issue the previous generation had not experienced. The impact of family pressures was compounded by, and in turn compounded, emerging health concerns.

It’s our age group isn’t it, people in their fifties … other people at work have the same issue, trying to care for parents when they’re getting to an age where their capacity is diminishing and they’re hitting their own health problems. [15]^2^

Competing demands on women’s time and attention took their toll on energy levels. Women described having to cope simultaneously with multiple scenarios:

It’s hectic, there always seems to be something going on. My partner lost his father a little while ago so we’ve been dealing with the house … Effectively, the boys, they’re three adults, and then with J, my partner, having things happening, and we’ve got the little one, it’s always just on the hop really. [2]

What with the kids and all their different issues and our own health issues and trying to keep the house and work. And then obviously parents now as well, they’ve become an issue where they weren’t before, they could help and support, and now we’ve lost one parent that was helpful and supportive and we’ve got others to care for. At this age, you’re in the middle and you’ve got kids at one end and elderly parents that are a bit like kids at the other end.

And in terms of your sexual relationship, has that changed?

Yes it has lately, but that’s through health and tiredness, both of us are tired. That plays a big part. [3]

Like a lot of teenagers, they don’t tend to help a lot … they’re still dependent on me for lifts and things like that so … yes, just a busy time and I think just having to do everything, with the house and the garden and, you know, I’ve got an elderly mum and I’ve got the kids, there’s lots of people demanding you. [4]

Among single women, the pressures tended to be work-related, limiting opportunities for dating.

…you’re busy, busy, busy all day, you’re out with work colleagues in the evening and the only time you get to yourself is literally the five or six hours you get to sleep [16]

Fatigue was a recurring theme throughout the interviews; women were simply exhausted. These were accounts of lives so busy that they left little time or energy to enjoy a regular and satisfying sex life.

We’re just tired, I’d rather sit in front of the telly with a glass of wine and fall asleep in the armchair, that’s us. [13]

So all those things conspire and then I suppose you could say that perhaps we never probably had massive libidos in the first place, and now I’m too tired. There’s been a lot going on and it just hasn’t been important. [18]

I’m, like, no, can’t be bothered I’ve got to work at night. I’m too tired, and he’s like that too. It disnae bother me at all. It seems to bother him mair than me. [11]

Against this backcloth, the link in the survey data between women’s sexual experience and their partners’ employment status – such that the more likely he was to be in paid work, the more likely they were to be sexually active, and the longer his working hours, the lower the likelihood of sexual dissatisfaction and poorer function – may seem counter-intuitive. Yet women whose partners were unemployed or in insecure jobs found it easy to make the link. In three instances, their partner’s job loss had directly coincided with either the onset of sexual difficulties, or their aggravation. One attributed her partner’s lack of sexual interest directly to more general insecurities:

I think his fear of failure, financially, you know, we are as I said less than secure despite this beautiful house, huge mortgage, never going to be repaid, no pension. That worries him, he can’t cope. It’s classic really … if he hits any sort of professional hiccup or he feels challenged, it’s not that he doesn’t enjoy sex but that he hides, he literally crawls under the duvet, he can sleep all weekend, he cannot get out of bed. [8]

## Discussion

Data from this population–based, cross-sectional survey of sexual attitudes and lifestyles, and from follow-up in-depth interviews, paint a complex picture of women’s sexual life-styles in middle age. The survey data show that over a third of women in midlife had been sexually inactive in the past month, yet less than half that proportion were dissatisfied with their sex lives. The survey data also suggest that the quality and frequency of sexual activity in midlife owes more to life stage and life events than to physiological aspects of aging or menopausal phase. Data from the indepth interviews concur with this finding; aside from the increasing prevalence of health conditions, the significance of age was construed by women themselves more in terms of social aspects of midlife than physiological decline or hormonal status. The strong and independent association in the survey data between all dimensions of sexual experience and aspects of personal relationships, particularly the ability to talk about sex, was also confirmed in women’s accounts. What emerged from the qualitative data as the most salient factor influencing women’s sexual activity in midlife was, however, the complexity of demands on women’s time in this period, and their impact on vitality.

Comparisons of our findings with those of others are limited by methodological differences. Where valid comparisons are possible, they reveal both continuities and divergences. Others have similarly reported less dissatisfaction than might be expected from levels of sexual inactivity (Trompeter et al., 2012), prompting the suggestion that women adjust their expectations of sexual frequency in midlife (Addis et al., 2006; Syme et al., 2019). Our finding that menopausal status exerts little independent effect on sexual activity, satisfaction, or function is consistent with those of other population-based surveys (Addis et al., 2006; Avis et al., 2018; Burghardt et al., 2020; Ringa et al., 2013). So, too, are our findings of independent associations between lower sexual function and poorer mental health (Gracia et al., 2018; Jackson et al., 2019) and between sexual dissatisfaction and higher educational attainment (Addis et al., 2006), reflecting, perhaps, higher expectations among more highly educated women. The powerful interplay between relationship quality and sexual satisfaction seen in our data and between sexual satisfaction and quality of life has been reported in many studies (Burleson et al., 2007; Davison et al., 2009; Harder et al., 2019; Jackson et al., 2019; Macleod et al., 2020). Our data illuminate the association between satisfaction with the sexual experience and happiness in the relationship, suggesting it to be bi-directional.

The finding from the qualitative component of our research, of the host of midlife stressors conspiring to limit the amount of time and energy available for sexual expression, is less well documented. An exception is the reference by Dennerstein et al. (2008) to “daily hassles” impacting on sexual activity. The finding is, however, consistent with evidence from extensive research carried out in recent decades on the relationship between age and wellbeing more generally. Attempts to chart fluctuations in well-being over the life course describe a U-shaped curve with the lowest levels occurring at ages 45–54 years (Blanchflower & Oswald, 2008; López-Ulloa et al., 2013; Steptoe et al., 2015; Wunder et al., 2013). Explanations for the midlife nadir (Bianchi, 2011) reflect scenarios described by many of the women we interviewed – the challenge of the work-life balance and the exacting and competing demands of family life, the burden of which has been shown to fall unequally on women (Bowman et al., 2013; Craig & Brown, 2017).

Such pressures are likely to have increased in recent times. This is the cohort of women, living through middle age at the beginning of the 21^st^ century, for whom demographic trends toward later marriage and childbearing, coupled with increased longevity, have increased the likelihood of simultaneously supporting both dependent children and elderly parents in midlife (Wiemers et al., 2015), while possibly coping with emerging health issues, earning them the sobriquet “the sandwich generation” (Brenna, 2020; Grundy & Henretta, 2006). It is also the cohort who reached midlife just after the 2008 economic recession, increasing the likelihood of financial insecurity and that of having grown children living at home. Further, this generation of women, born in the 1950s and 60s, is the first for whom it was commonplace to combine work outside and inside the home. Generational declines in the frequency of sexual activity have been reported in several Western-style countries in recent decades, particularly among middle-aged women (Burghardt et al., 2020; Twenge et al., 2017; De Visser et al., 2014; Wellings et al., 2019), and it seems plausible that the increasing “busyness” of life might feature among contributing factors. This interpretation resonates with the heteronormative hypotheses advanced by van Anders et al. (2022). A possible pathway by which heteronormativity might influence sexual experience in women partnered with men, it is suggested, is via inequitable gendered divisions of household labor and the care of relationships with partners, children, and elderly parents (van Anders et al., 2022).

A strength of this study is its focus on multiple dimensions of sexual experience at midlife, the inclusion of a wide range of indicators of possible determinants, and the mixed methods design. We found no other study that has combined both quantitative and qualitative methods of investigation to explore the relationship between health and lifestyle factors and dimensions of sexual experience. As intended, the findings of the qualitative component were invaluable in compensating for some of the short-comings of a cross-sectional survey. They helped in tracing likely pathways in the associations found in the survey data, for example, from unemployment via depression and low self-esteem to sexual activity. They helped establish the causal direction in associations, the two-way relationship between relationship quality and sexual wellbeing, for example, and between mental health and sexual satisfaction. Perhaps most importantly, they shed light on what women themselves see as the most significant influences on their sexual experience in midlife.

By restricting the study sample to women, we have, however, limited insights into gendered sexual relations in midlife. Excluding women who had only ever had sex with a woman also limits the utility of the findings. A further limitation of the study relates to the interval since the data were collected and hence our inability to assess how subsequent events have influenced women’s sexual experience. We cannot rule out the effect of the researcher on women’s responses though this may have been somewhat mitigated by the wide age range of interviewers, from those in their 30s to those in their 60s.

The findings of this study have implications for the conduct of research in this area. The qualitative research highlighted limitations of the survey data in meeting the study objectives. The fact that so few variables were independently associated with dimensions of sexual experience may be partly explained by the interaction between the independent variables – between relationship status and health, for example, unemployment and depression, and socio-economic status and BMI. Women themselves found it difficult to disentangle the variables. But the limited number of independent associations also reflects the fact that no indicators in the Natsal-3 questionnaire adequately captured the factors seen by women themselves as most strongly influencing sexual frequency and satisfaction in middle age, notably tiredness consequent on the pressures of midlife. Fatigue is often attributed narrowly to menopausal status (Kong et al., 2019) yet more than a quarter of a century ago it was authoritatively established that, independently of hormonal status, well-being was an important predictor of positive sexual experience and the best predictor of well-being was tiredness (Cawood & Bancroft, 1996). The link made by women themselves between energy levels and sexual dissatisfaction seen in our own research and that of others (Davison et al., 2009; Prairie et al., 2015) argues for the inclusion of validated measures of fatigue, stress, and sleep deprivation (Devine et al., 2005; Nielsen et al., 2016; Valko et al., 2008), in surveys of sexual lifestyles. The consequences of fatigue and sleep deprivation have been described for a number of health outcomes in midlife, including susceptibility to colds, smoking rates, and poorer cognitive function (Scott et al., 2013) but rarely for sexual activity.

In the context of policy and practice, our data may inform the design of effective interventions aimed at improving the sexual wellbeing of women in midlife. The evidence from women’s accounts of reciprocal links between relationship quality and sexual satisfaction and between sexual activity and general well-being shown in other research (Burleson et al., 2007; Skałacka & Gerymski, 2019) suggest that the benefits extend beyond sexual wellbeing.

Our data also suggest that satisfaction may be a better end point than function or frequency for effective intervention. The lower prevalence of sexual dissatisfaction compared with that of sexual inactivity indicates that what is important is not how often women have sex, but whether it matters to them. As we and others have found, sexual satisfaction depends as much on intimacy and closeness as on sexual frequency (Erens et al., 2014; Schoenfeld et al., 2017). This is an important point to convey especially as what evidence there is suggests a widespread belief among individuals that others have sex more frequently than they do themselves (Ipsos-MORI, 2011). Providing reliable information may lead to more realistic expectations and so contribute to higher satisfaction levels.

Our findings caution against focusing narrowly on the proximate determinants of sexual expression and emphasize the importance of taking account of the wider social context of women’s lives in addressing issues relating to sexual wellbeing. The quality of sexual experience needs to be evaluated against a backcloth of the social and historical forces shaping it. In this respect, population-level data on sexual behavior serve a useful purpose. Establishing normative ranges may provide reassurance to women that their sexual behavior is “normal for now” in terms of both time of life and life in our times.

## Figures and Tables

**Table 1:** Prevalence of sexual dissatisfaction, sexual inactivity and lower sexual function among women aged 40–59, by selected variables.

	Sexual dissatisfaction				No heterosexual sex in last 4 weeks				Lower sexual function		
	unweighted/weighted bases	yes			unweighted/weighted bases				unweighted/weighted bases		
		%	CI			%	CI			%	CI
**ALL**	**2071, 2648**	**16.2%**	**14.6–18.0**		**2077, 2654**	**35.6**	**33.4–37.9**		**1655, 2239**	**23.3**	**21.2–25.6**
**Age group**											
40–44	569, 718	16.2%	(13.2–19.6)		571, 720	27.1%	(23.5–30.9)		502, 659	21.0%	(17.4–25.1)
45–49	565, 737	17.5%	(14.4–21.0)		563, 735	31.7%	(27.8–35.8)		481, 655	23.6%	(19.7–28.0)
50–54	476, 613	14.2%	(11.1–18.0)		477, 612	35.1%	(30.7–39.7)		370, 511	21.3%	(17.1–26.3)
55–59	461, 580	16.7%	(13.5–20.6)		466, 587	51.6%	(46.6–56.6)		302, 414	28.7%	(23.6–34.5)
**HORMONAL**											
**Menopausal status**											
pre-menopausal	871, 1105	16.7%	(14.3–19.6)		874, 1108	27.2%	(24.2–30.4)		769, 1008	21.3%	(18.3–24.6)
peri-menopausal	146, 199	15.1%	(9.9–22.3)		143, 195	33.1%	(25.4–41.8)		124, 178	26.5%	(18.9–35.9)
post-menopausal, no HRT	945, 1205	15.5%	(13.3–18.1)		949, 1210	43.4%	(39.9–46.8)		679, 938	24.2%	(21.0–27.9)
post-menopausal, HRT	88, 110	19.6%	(12.0–30.4)		90, 112	40.2%	(29.9–51.5)		66, 89	33.6%	(22.4–46.8)
**HEALTH STATUS**											
**own health (self-reported)**											
good/very good	1594, 2091	15.4%	(13.6–17.3)		1592, 2086	31.2%	(28.9–33.6)		1333, 1828	20.6%	(18.4–23.1)
fair/bad/very bad	477, 557	19.4%	(15.9–23.3)		485, 568	51.8%	(46.7–56.9)		322, 412	35.1%	(29.7–40.8)
**Screen positive for current depression**^#^ **(last 2 w)**											
no	1782, 2319	14.4%	(12.8–16.3)		1777, 2309	33.4%	(31.1–35.8)		1451, 1988	19.8%	(17.7–22.1)
yes	286, 326	28.9%	(23.7–34.8)		290, 331	50.1%	(43.7–56.5)		203, 250	51.1%	(43.2–58.9)
**BMI**											
under/normal	900, 1161	13.4%	(11.2–15.9)		900, 1158	31.1%	(28.0–34.3)		751, 1016	20.8%	(17.8–24.1)
overweight/obese	1067, 1347	18.5%	(16.2–21.0)		1074, 1357	39.4%	(36.3–42.6)		829, 1112	25.9%	(23.0–29.1)
**RELATIONSHIP STATUS**											
**Most recent sexual relationship**											
cohabiting	1428, 2057	14.8%	(13.0–16.8)		1436, 2066	30.5%	(28.1–33.1)		1202, 1815	24.0%	(21.6–26.6)
steady	366, 340	15.7%	(12.1–20.0)		366, 340	44.5%	(39.0–50.1)		278, 268	13.6%	(9.9–18.3)
previously steady	61, 51	28.5%	(18.5–41.3)		61, 51	72.2%	(58.5–82.7)		44, 35	34.8%	(21.1–51.5)
other	197, 176	32.4%	(25.4–40.3)		197, 176	66.8%	(58.3–74.3)		128, 118	31.4%	(22.7–41.6)
**Satisfaction with relationship**											
happy (grouped)	830, 1217	8.0%	(6.3–10.2)		828, 1214	14.8%	(12.5–17.5)		830, 1217	16.3%	(13.9–19.1)
not happy (grouped)	405, 584	19.6%	(15.9–24.0)		405, 584	21.8%	(17.7–26.4)		405, 584	32.8%	(28.0–37.9)
**Talking about sex with partner**											
always easy	420, 551	10.1%	(7.3–13.7)		422, 553	26.9%	(22.6–31.7)		356, 493	10.1%	(7.0–14.2)
sometimes easy, sometimes difficult	1456, 1855	15.7%	(13.8–17.8)		1456, 1854	36.0%	(33.4–38.7)		1180, 1582	24.2%	(21.7–26.9)
always difficult	170, 212	38.1%	(30.3–46.4)		171, 213	52.5%	(44.5–60.4)		113, 155	56.7%	(46.6–66.2)
**ATTITUDES**											
**Natural to want less sex with age**											
agree	935, 1224	15.2%	(13.0–17.7)		936, 1224	38.7%	(35.5–42.0)		746, 1036	27.7%	(24.5–31.2)
don’t agree	1107, 1391	17.3%	(15.0–19.8)		1110, 1395	32.9%	(30.0–35.9)		889, 1181	19.5%	(16.8–22.5)
**Men have higher sex drive than women**											
agree	1012, 1317	15.7%	(13.5–18.1)		1017, 1323	33.6%	(30.6–36.7)		831, 1146	25.9%	(22.8–29.3)
don’t agree	1039, 1309	16.7%	(14.4–19.3)		1038, 1306	37.4%	(34.3–40.7)		812, 1078	20.6%	(17.8–23.8)
**LIFESTYLE**											
**Quintile of adjusted IMD^∞^ (for Great Britain)**											
1 [least deprived]	467, 627	16.0%	(12.8–19.7)		463, 621	28.9%	(24.8–33.4)		400, 557	24.4%	(20.1–29.3)
2	438, 573	14.9%	(11.7–18.8)		440, 575	33.5%	(29.0–38.4)		356, 495	21.1%	(16.8–26.1)
3	409, 504	18.1%	(14.5–22.4)		408, 500	34.2%	(29.5–39.3)		320, 418	22.8%	(18.3–28.1)
4	381, 485	14.5%	(11.2–18.5)		383, 488	40.7%	(35.4–46.2)		295, 398	24.9%	(20.0–30.5)
5 [most deprived]	376, 459	17.9%	(14.0–22.5)		383, 470	43.2%	(37.9–48.7)		284, 372	23.3%	(18.6–28.8)
**Educated to degree-level**											
no	1146, 1500	15.2%	(13.1–17.5)		1148, 1503	33.4%	(30.6–36.4)		944, 1297	22.5%	(19.8–25.4)
yes	532, 669	21.7%	(18.1–25.7)		534, 670	34.3%	(30.1–38.8)		421, 562	23.8%	(19.5–28.6)
**Paid employment (last week)**											
employed	1484, 1949	16.1%	(14.3–18.2)		1488, 1954	31.8%	(29.4–34.2)		1237, 1703	22.4%	(20.0–25.1)
unemployed	529, 630	17.0%	(13.9–20.6)		530, 631	45.2%	(40.3–50.2)		386, 494	25.8%	(21.5–30.7)
retired	58, 68	11.0%	(5.5–21.0)		59, 69	56.5%	(42.5–69.6)		32, 42	27.7%	(14.1–47.3)
**Hours worked per week¥**											
50 or more	101, 133	13.4%	(8.2–21.0)		102, 135	29.4%	(21.2–39.3)		88, 121	18.9%	(11.8–28.9)
35 to 49	650, 824	17.3%	(14.4–20.6)		653, 829	34.2%	(30.6–38.0)		525, 706	21.9%	(18.4–25.9)
less than 35	732, 990	15.3%	(12.8–18.3)		730, 986	30.2%	(26.9–33.8)		623, 873	23.1%	(19.7–26.9)
**Partner’s paid employment (last week)**											
employed	1032, 1597	13.3%	(11.3–15.6)		1028, 1591	20.5%	(18.1–23.3)		981, 1523	23.0%	(20.3–25.8)
unemployed	105, 160	17.1%	(10.9–25.7)		105, 159	44.3%	(34.6–54.5)		90, 139	25.9%	(17.7–36.2)
retired	93, 142	15.7%	(9.4–25.3)		94, 142	42.9%	(32.6–53.9)		73, 111	29.6%	(20.0–41.6)
**Hours worked per week by partner¥**											
50 or more	203, 320	14.0%	(9.7–19.8)		202, 318	29.9%	(12.7–23.3)		192, 303	24.1%	(18.3–31.0)
35 to 49	722, 1114	11.9%	(9.7–14.6)		721, 1114	20.3%	(17.3–23.6)		694, 1075	21.3%	(18.3–24.7)
less than 35	102, 154	21.9%	(14.7–31.2)		100, 152	17.4%	(21.2–40.2)		90, 137	33.0%	(23.7–43.8)
**LIFESTAGE**											
**Age of youngest child (excluding adopted)**											
no children	346, 352	19.9%	(15.9–24.7)		347, 353	48.9%	(43.2–54.6)		228, 248	21.9%	(16.8–28.0)
0–5	86, 101	27.1%	(18.3–38.2)		87, 102	30.6%	(21.7–41.2)		78, 94	29.4%	(19.7–41.4)
6–11	356, 451	18.5%	(14.6–23.2)		354, 449	25.6%	(21.3–30.5)		317, 418	21.7%	(17.1–27.0)
12–19	513, 771	14.9%	(12.0–18.4)		515, 773	30.1%	(26.1–34.4)		436, 680	23.0%	(19.0–27.6)
20+	758, 960	13.8%	(11.4–16.5)		760, 963	40.4%	(36.7–44.2)		589, 791	24.4%	(20.8–28.3)

¥ Base = those in current employment;

# In last two weeks;

∞ IMD for Britain

**Table 2:** Age-adjusted odds ratios for sexual dissatisfaction, sexual inactivity, and lower sexual function among women, by selected variables.

	Sexual dissatisfaction					No heterosexual sex in last 4 weeks					Lower sexual function			
	unwt, wt bases	age-adjusted				unwt, wt bases	age-adjusted				unwt, wt bases	age-adjusted		
		OR	Cl	p-val			OR	Cl	p-val			OR	Cl	p-val
**ALL**	**2071, 2648**					**2077, 2654**					**1655, 2239**			
**HORMONAL**														
**Menopausal status**				0.6854					0.9463					0.2832
pre-menopausal	871, 1105	1.00	-			874, 1108	1.00	-			769, 1008	1.00	-	
peri-menopausal	146, 199	0.85	(0.49–1.48)			143, 195	1.02	(0.66–1.56)			124, 178	1.2	(0.72–2.01)	
post-menopausal, no HRT	945, 1205	0.83	(0.50–1.38)			949, 1210	1.10	(0.76–1.59)			679, 938	0.93	(0.57–1.51)	
post-menopausal, HRT	88, 110	1.13	(0.54–2.35)			90, 112	1.14	(0.67–1.94)			66,89	1.57	(0.80–3.05)	
**HEALTH STATUS**														
**own health (self-reported)**				0.0376					<0.0001					<0.0001
good/very good	1594, 2091	1.00	-			1592, 2086	1.00	-			1333, 1828	1.00		
fair/bad/very bad	477, 557	1.34	(1.02–1.77)			485, 568	2.11	(1.67–2.67)			322, 412	2.01	(1.51–2.69)	
**Screen positive for current depression^#^ (last 2 w)**				<0.0001					<0.0001					<0.0001
no	1782, 2319	1.00	-			1777, 2309	1.00	-			1451, 1988	1.00	-	
yes	286, 326	2.42	(1.78–3.28)			290, 331	2.07	(1.56–2.75)			203, 250	4.27	(3.02–6.04)	
**BMI**				0.0122					0.0002					0.0268
under/normal	900, 1161	1.00	-			900, 1158	1.00	-			751, 1016	1.00		
overweight/obese **RELATIONSHIP STATUS**	1067, 1347	1.47	(1.14–1.91)			1074, 1357	1.38	(1.13–1.68)			829, 1112	1.32	(1.03–1.68)	
**Most recent sexual relationship**				<0.0001					<0.0001					0.0002
cohabiting	1428, 2057	1.00	-			1436, 2066	1.00	-			1202, 1815	1.00	-	
steady	366, 340	1.07	(0.77–1.50)			366, 340	1.92	(1.48–2.48)			278, 268	0.50	(0.34–0.74)	
previously steady	61,51	2.30	(1.31–4.05)			61,51	6.74	(3.70–12.29)			44, 35	1.77	(0.89–3.53)	
other	197, 176	2.76	(1.91–4.00)			197, 176	5.19	(3.54–7.59)			128, 118	1.51	(0.95–2.42)	
**Satisfaction with relationship**				<0.0001					0.0046					<0.0001
happy (grouped)	830, 1217	1.00	-			828, 1214	1.00	-			830, 1217	1.00		
not happy (grouped)	405, 584	2.82	(1.94–4.09)			405, 584	1.59	(1.15–2.19)			405, 584	2.48	1.86–3.32	
**Talking about sex with partner**				<0.0001					<0.0001					<0.0001
always easy	420, 551	1.00	-			422, 553	1.00	-			356, 493	1.00	-	
sometimes easy, sometimes difficult	1456, 1855	1.66	(1.13–2.42)			1456, 1854	1.59	(1.22–2.07)			1180, 1582	2.98	1.94–4.58	
always difficult	170, 212	5.48	(3.37–8.90)			171, 213	3.16	(2.13–4.70)			113, 155	11.92	6.88–20.65	
**ATTITUDES**														
**Natural to want less sex with age**				0.2219					0.0646					0.0006
agree	935, 1224	1.00	-			936, 1224	1.00	-			746, 1036	1.00	-	
don’t agree	1107, 1391	1.17	(0.91–1.50)			1110, 1395	0.83	(0.69–1.01)			889, 1181	0.65	(0.50–0.83)	
**Men have higher sex drive than women**				0.5589					0.0463					0.0264
agree	1012, 1317	1.00	-			1017, 1323	1.00	-			831, 1146	1.00	-	
don’t agree **LIFESTYLE**	1039, 1309	1.08	(0.84–1.38)			1038, 1306	1.22	(1.00–1.49)			812, 1078	0.75	(0.58–0.97)	
**Quintile of adjusted IMD^∞^ (For Great Britain)**				0.5662					0.0001					0.8337
1 [least deprived]	467, 627	1.00	-			463, 621	1.00	-			400, 557	1.00	-	
2	438, 573	0.92	(0.63–1.36)			440, 575	1.28	(0.95–1.73)			356, 495	0.83	(0.57–1.20)	
3	409, 504	1.16	(0.81–1.68)			408, 500	1.30	(0.96–1.76)			320,418	0.92	(0.63–1.32)	
4	381,485	0.89	(0.60–1.31)			383, 488	1.81	(1.32–2.48)			295, 398	1.02	(0.70–1.49)	
5 [most deprived]	376, 459	1.14	(0.78–1.68)			383, 470	2.00	(1.46–2.72)			284, 372	0.94	(0.65–1.38)	
**Educated to degree-level**				0.0023					0.7755					0.6306
no	1146, 1500	1.00	-			1148, 1503	1.00	-			944, 1297	1.00	-	
yes	532, 669	1.54	(1.17–2.04)			534, 670	1.04	(0.83–1.31)			421,562	1.08	(0.80–1.45)	
**Paid employment (last week)**				0.4770					<0.0001					0.3653
employed	1484, 1949	1.00	-			1488, 1954	1.00	-			1237, 1703	1.00	-	
unemployed	529, 630	1.06	(0.81–1.40)			530, 631	1.69	(1.35–2.13)			386, 494	1.20	(0.91–1.59)	
retired	58, 68	0.65	(0.30–1.38)			59, 69	1.82	(1.03–3.21)			32, 42	1.33	(0.57–3.07)	
**Hours worked per week¥**				0.4610					0.2201					0.6137
50 or more	101, 133	1.00				102, 135	1.00				88, 121	1.00		
35 to 49	650, 824	1.36	(0.76–2.42)			653, 829	1.32	0.82–2.12			525, 706	1.24	(0.69–2.23)	
less than 35	732, 990	1.18	(0.66–2.10)			730, 986	1.09	0.68–1.74			873, 623	1.33	(0.74–2.38)	
**Partner’s paid employment (last week)**				0.5425					<0.0001					0.4051
employed	1032, 1597	1.00	-			1028, 1591	1.00	-			981, 1523	1.00	-	
unemployed	105, 160	1.34	0.77–2.31			105, 159	3.00	(1.91–4.69)			90, 139	1.17	(0.71–1.94)	
retired	93, 142	1.18	0.61–2.30			94, 142	2.00	(1.23–3.26)			73, 111	1.41	(0.82–2.43)	
**Hours worked per week by partner¥**				0.0353					0.1554					0.0960
50 or more	203, 320	1.00	-			202, 318	1.00	-			192, 303	1.00		
35 to 49	722, 1114	0.83	(0.51–1.35)			721,1114	1.23	(0.81–1.86)			694, 1075	0.86	(0.57–1.28)	
less than 35	102, 154	1.70	(0.89–3.22)			100, 152	1.78	(0.99–3.20)			90, 137	1.47	(0.82–2.63)	
**LIFESTAGE**														
**Age of youngest child (excluding adopted)**				0.0059					<0.0001					0.4150
no children	346, 352	1.00				347, 353	1.00	-			228, 248	1.00		
0–5	86, 101	1.76	(0.97–3.18)			87, 102	0.74	(0.43–1.29)			78,94	1.81	(0.96–3.44)	
6–11	356, 451	1.01	(0.67–1.54)			354, 449	0.49	(0.34–0.69)			317, 418	1.11	(0.70–1.76)	
12–19	513, 771	0.73	0.51–1.05			515, 773	0.49	(0.36–0.67)			436, 680	1.10	(0.73–1.64)	
20+	758, 960	0.58	0.40–0.86			760, 963	0.52	(0.38–0.70)			589, 791	0.96	(0.63–1.45)	

¥ Base = those in current employment;

# In last two weeks;

∞ IMD for Britain

**Table 3:** Fully adjusted odds for sexual dissatisfaction, low sexual frequency, and lower sexual function among women.

	Sexual dissatisfaction				No heterosexual sex in last 4 weeks				Lower sexual function		
	OR	CI	p-val		OR	CI	p-val		OR	CI	p-val
**Own health (self-reported)**											0.0431
good/very good									1.00		
fair/bad/very bad									1.53	(1.01–2.32)	
**Screen positive for current depression (last 2 w)**											<0.0001
no									1.00	-	
yes									3.55	(2.21–5.70)	
**Satisfaction with relationship**			<0.0001				0.0050				0.0072
happy (grouped)	1.00	-			1.00	-			1.00	-	
not happy (grouped)	2.86	(1.88–4.35)			1.59	(1.15–2.19)			2.22	(1.62–3.04)	
**Talking about sex with partner**			0.0004								<0.0001
always easy	1.00	-							1.00	-	
sometimes easy, sometimes difficult	1.52	(0.83–2.77)							3.18	(1.83–5.50)	
always difficult	4.39	(2.03–9.50)							12.30	(6.18–24.49)	
**Educated to degree-level**			0.0069								
no	1.00	-									
yes	1.74	(1.17–2.61)									
**Age of youngest child (excluding adopted)**			0.0449								
no children	1.00	-									
0–5	3.61	(1.40–9.29)									
6–11	2.36	(1.12–4.96)									
12–19	1.65	(0.79–3.42)									
20+	1.07	(0.48–2.40)									

Age and menopausal status included a priori; all significant variables from Table 2 included in the model. FINAL MODEL – all significant variables were adjusted for each other, after which those that remained significant were put into final model (age and menopausal status included a priori).

# In last two weeks

**Table 4:** Characteristics of women interviewed in-depth as provided in interviews.

Number in text	Age	Relationship status at the time of interview	Menopausal stage
1 RL	54	Married, about to separate	Menopausal
2 KM	48	Cohabiting	Menopausal
3 RL	53	Married	Menopausal
4 KM	54	Divorced	Menopausal
5 KM	48	Cohabiting after being twice married	Pre-menopausal
6 JD	47	Separated	Pre-menopausal
7 JD	59	Married	Post-menopausal
8 JD	55	Married	Post-menopausal
9 JD	58	Cohabiting	Post-menopausal
10 JD	49	No current relationship	Menopausal
11 RL	47	Cohabiting	Menopausal
12 KW	48	No current relationship	Pre-menopausal
13 RL	50	Married	Unsure (using Mirena)
14 KW	57	Divorced; in non-cohabiting relationship	Post-menopausal
15 KM	57	Mardried	Post-menopausal
16 JD	49	Single	Unsure (using Mirena)
17 KW	51	Married, with extramarital partner	Menopausal
18 JD	59	Married	Post-menopausal
19 JD	51	Non-cohabiting partner	Menopausal
20 KW	48	Single	Pre-menopausal
21 RL	48	Separated	Menopausal
22 RL	46	Separated	Menopausal
23 RL	47	Separated, about to remarry	Menopausal

Initials are those of the interviewer in each case

## Footnotes

1 aOR = age-adjusted Odds Ratio; AOR = fully adjusted Odds Ratio

2 Numbers following quotes in the text refer to respondents identified in Table 4
